# Supplementary material for: ProteinShader: illustrative rendering of macromolecules
Source: BMC Struct Biol. 2009 Mar 30;9:19. doi: 10.1186/1472-6807-9-19 (PMC2672931; doi:10.1186/1472-6807-9-19)
Supplement: Additional file 1 — ProteinShader program without source code. This compressed file contains the complete ProteinShader program including associated libraries, but no source code. A README.txt file gives an overview of the ProteinShader distribution, and the index.html file in the help subdirectory has directions on getting started with the program as well as a set of tutorials. [file 1472-6807-9-19-S1.zip › ProteinShader-beta-0_9_4-binary/help/api/org/proteinshader/gui/components/controlpanel/OptimizePanel.html]

OptimizePanel (ProteinShader API)


|  |  |  |
| --- | --- | --- |
| **Overview** \| **Package** \| **Class** \| **Use** \| **Tree** \| **Deprecated** \| **Index** \| **Help** | |  |
| **PREV CLASS**   **NEXT CLASS** | **FRAMES**    **NO FRAMES**     **All Classes** |  |
| SUMMARY: NESTED \| FIELD \| CONSTR \| METHOD | DETAIL: FIELD \| CONSTR \| METHOD |  |


---


## org.proteinshader.gui.components.controlpanel Class OptimizePanel

```
java.lang.Object
  java.awt.Component
      java.awt.Container
          javax.swing.JComponent
              javax.swing.JPanel
                  org.proteinshader.gui.components.controlpanel.OptimizePanel
```

**All Implemented Interfaces:**: ImageObserver, MenuContainer, Serializable, Accessible

---

``` public class OptimizePanel extends JPanel ```

This control panel allows the user to optimize the display for image
quality or rendering speed.

**See Also:**: Serialized Form

---

**Nested Class Summary**

**Nested classes/interfaces inherited from class javax.swing.JPanel**

`JPanel.AccessibleJPanel`

**Nested classes/interfaces inherited from class javax.swing.JComponent**

`JComponent.AccessibleJComponent`

**Nested classes/interfaces inherited from class java.awt.Container**

`Container.AccessibleAWTContainer`

**Nested classes/interfaces inherited from class java.awt.Component**

`Component.AccessibleAWTComponent, Component.BltBufferStrategy, Component.FlipBufferStrategy`


| **Field Summary** | |
| --- | --- |
| `static String` | `MENU_NAME`             The menu name "Tiling" will be returned by toString(). |
| `static int` | `SPINNER_MAX`             The maximum value in a spinner is 100. |
| `static int` | `SPINNER_MIN`             The minimum value in a spinner is 3 (same as Sphere.MIN\_TILING). |

**Fields inherited from class javax.swing.JComponent**

`accessibleContext, listenerList, TOOL_TIP_TEXT_KEY, ui, UNDEFINED_CONDITION, WHEN_ANCESTOR_OF_FOCUSED_COMPONENT, WHEN_FOCUSED, WHEN_IN_FOCUSED_WINDOW`

**Fields inherited from class java.awt.Component**

`BOTTOM_ALIGNMENT, CENTER_ALIGNMENT, LEFT_ALIGNMENT, RIGHT_ALIGNMENT, TOP_ALIGNMENT`

**Fields inherited from interface java.awt.image.ImageObserver**

`ABORT, ALLBITS, ERROR, FRAMEBITS, HEIGHT, PROPERTIES, SOMEBITS, WIDTH`


**Constructor Summary**

`OptimizePanel(Mediator mediator,
Frame dialogOwner)`
  
          Constructs an OptimizePanel.


| **Method Summary** | |
| --- | --- |
| `void` | `addAllOtherComponentsToCenterPanel()`             This helper method for the constructor adds the radio buttons and the tiling spinner to the center panel. |
| `void` | `addAutoTilingButtonPanelToNorth()`             This helper method for the constructor adds a check box button for turning automatic tiling (level of detail) on/off. |
| `void` | `applyDefaultTilingNumber()`             Applies the default number tiling number (slices and/or stacks) for the currently selected geometric object. |
| `void` | `applySpinnerTilingNumber()`             Applies the tiling number in the spinner to the currently selected geometric object. |
| `void` | `setCenterPanelComponentsEnabled(boolean enabled)`             Setting enabled to false will cause the center panel components to be grayed out (so they cannot be used). |
| `void` | `setSpinnerToCylinderCapValue()`             Sets the value in the tiling spinner to the value displayed under the radio button for cylinder caps. |
| `void` | `setSpinnerToCylinderValue()`             Sets the value in the tiling spinner to the value displayed under the radio button for cylinders. |
| `void` | `setSpinnerToSphereValue()`             Sets the value in the tiling spinner to the value displayed under the radio button for spheres. |
| `String` | `toString()`             Returns a name suitable for use in a menu. |
| `void` | `updateCylinderCapsLabel(int tiling)`             Updates the tiling number text label below the 'Cylinder Caps' combo box menu, updates the spinner value to match, and also sets the 'Cylinder Caps' radio button as being selected. |
| `void` | `updateCylindersLabel(int tiling)`             Updates the tiling number text label below the 'Cylinders' combo box menu, updates the spinner value to match, and also sets the 'Cylinders' radio button as being selected. |
| `void` | `updateSpheresLabel(int tiling)`             Updates the tiling number text label below the "Spheres" combo box menu, updates the spinner value to match, and also sets the "Spheres" radio button as being selected. |

**Methods inherited from class javax.swing.JPanel**

`getAccessibleContext, getUI, getUIClassID, paramString, setUI, updateUI`

**Methods inherited from class javax.swing.JComponent**

`addAncestorListener, addNotify, addVetoableChangeListener, computeVisibleRect, contains, createToolTip, disable, enable, firePropertyChange, firePropertyChange, firePropertyChange, fireVetoableChange, getActionForKeyStroke, getActionMap, getAlignmentX, getAlignmentY, getAncestorListeners, getAutoscrolls, getBorder, getBounds, getClientProperty, getComponentGraphics, getComponentPopupMenu, getConditionForKeyStroke, getDebugGraphicsOptions, getDefaultLocale, getFontMetrics, getGraphics, getHeight, getInheritsPopupMenu, getInputMap, getInputMap, getInputVerifier, getInsets, getInsets, getListeners, getLocation, getMaximumSize, getMinimumSize, getNextFocusableComponent, getPopupLocation, getPreferredSize, getRegisteredKeyStrokes, getRootPane, getSize, getToolTipLocation, getToolTipText, getToolTipText, getTopLevelAncestor, getTransferHandler, getVerifyInputWhenFocusTarget, getVetoableChangeListeners, getVisibleRect, getWidth, getX, getY, grabFocus, isDoubleBuffered, isLightweightComponent, isManagingFocus, isOpaque, isOptimizedDrawingEnabled, isPaintingTile, isRequestFocusEnabled, isValidateRoot, paint, paintBorder, paintChildren, paintComponent, paintImmediately, paintImmediately, print, printAll, printBorder, printChildren, printComponent, processComponentKeyEvent, processKeyBinding, processKeyEvent, processMouseEvent, processMouseMotionEvent, putClientProperty, registerKeyboardAction, registerKeyboardAction, removeAncestorListener, removeNotify, removeVetoableChangeListener, repaint, repaint, requestDefaultFocus, requestFocus, requestFocus, requestFocusInWindow, requestFocusInWindow, resetKeyboardActions, reshape, revalidate, scrollRectToVisible, setActionMap, setAlignmentX, setAlignmentY, setAutoscrolls, setBackground, setBorder, setComponentPopupMenu, setDebugGraphicsOptions, setDefaultLocale, setDoubleBuffered, setEnabled, setFocusTraversalKeys, setFont, setForeground, setInheritsPopupMenu, setInputMap, setInputVerifier, setMaximumSize, setMinimumSize, setNextFocusableComponent, setOpaque, setPreferredSize, setRequestFocusEnabled, setToolTipText, setTransferHandler, setUI, setVerifyInputWhenFocusTarget, setVisible, unregisterKeyboardAction, update`

**Methods inherited from class java.awt.Container**

`add, add, add, add, add, addContainerListener, addImpl, addPropertyChangeListener, addPropertyChangeListener, applyComponentOrientation, areFocusTraversalKeysSet, countComponents, deliverEvent, doLayout, findComponentAt, findComponentAt, getComponent, getComponentAt, getComponentAt, getComponentCount, getComponents, getComponentZOrder, getContainerListeners, getFocusTraversalKeys, getFocusTraversalPolicy, getLayout, getMousePosition, insets, invalidate, isAncestorOf, isFocusCycleRoot, isFocusCycleRoot, isFocusTraversalPolicyProvider, isFocusTraversalPolicySet, layout, list, list, locate, minimumSize, paintComponents, preferredSize, printComponents, processContainerEvent, processEvent, remove, remove, removeAll, removeContainerListener, setComponentZOrder, setFocusCycleRoot, setFocusTraversalPolicy, setFocusTraversalPolicyProvider, setLayout, transferFocusBackward, transferFocusDownCycle, validate, validateTree`

**Methods inherited from class java.awt.Component**

`action, add, addComponentListener, addFocusListener, addHierarchyBoundsListener, addHierarchyListener, addInputMethodListener, addKeyListener, addMouseListener, addMouseMotionListener, addMouseWheelListener, bounds, checkImage, checkImage, coalesceEvents, contains, createImage, createImage, createVolatileImage, createVolatileImage, disableEvents, dispatchEvent, enable, enableEvents, enableInputMethods, firePropertyChange, firePropertyChange, firePropertyChange, firePropertyChange, firePropertyChange, firePropertyChange, getBackground, getBounds, getColorModel, getComponentListeners, getComponentOrientation, getCursor, getDropTarget, getFocusCycleRootAncestor, getFocusListeners, getFocusTraversalKeysEnabled, getFont, getForeground, getGraphicsConfiguration, getHierarchyBoundsListeners, getHierarchyListeners, getIgnoreRepaint, getInputContext, getInputMethodListeners, getInputMethodRequests, getKeyListeners, getLocale, getLocation, getLocationOnScreen, getMouseListeners, getMouseMotionListeners, getMousePosition, getMouseWheelListeners, getName, getParent, getPeer, getPropertyChangeListeners, getPropertyChangeListeners, getSize, getToolkit, getTreeLock, gotFocus, handleEvent, hasFocus, hide, imageUpdate, inside, isBackgroundSet, isCursorSet, isDisplayable, isEnabled, isFocusable, isFocusOwner, isFocusTraversable, isFontSet, isForegroundSet, isLightweight, isMaximumSizeSet, isMinimumSizeSet, isPreferredSizeSet, isShowing, isValid, isVisible, keyDown, keyUp, list, list, list, location, lostFocus, mouseDown, mouseDrag, mouseEnter, mouseExit, mouseMove, mouseUp, move, nextFocus, paintAll, postEvent, prepareImage, prepareImage, processComponentEvent, processFocusEvent, processHierarchyBoundsEvent, processHierarchyEvent, processInputMethodEvent, processMouseWheelEvent, remove, removeComponentListener, removeFocusListener, removeHierarchyBoundsListener, removeHierarchyListener, removeInputMethodListener, removeKeyListener, removeMouseListener, removeMouseMotionListener, removeMouseWheelListener, removePropertyChangeListener, removePropertyChangeListener, repaint, repaint, repaint, resize, resize, setBounds, setBounds, setComponentOrientation, setCursor, setDropTarget, setFocusable, setFocusTraversalKeysEnabled, setIgnoreRepaint, setLocale, setLocation, setLocation, setName, setSize, setSize, show, show, size, transferFocus, transferFocusUpCycle`

**Methods inherited from class java.lang.Object**

`clone, equals, finalize, getClass, hashCode, notify, notifyAll, wait, wait, wait`

**Field Detail**

### MENU\_NAME

```
public static final String MENU_NAME
```

:   The menu name "Tiling" will be returned by toString().

    **See Also:**: Constant Field Values

---


### SPINNER\_MIN

```
public static final int SPINNER_MIN
```

:   The minimum value in a spinner is
    3 (same as Sphere.MIN\_TILING).

    **See Also:**: Constant Field Values

---


### SPINNER\_MAX

```
public static final int SPINNER_MAX
```

:   The maximum value in a spinner is 100.

    **See Also:**: Constant Field Values


**Constructor Detail**

### OptimizePanel

```
public OptimizePanel(Mediator mediator,
                     Frame dialogOwner)
```

:   Constructs an OptimizePanel.

    **Parameters:**: `mediator` - the centralized Mediator that most listeners need to call on to accomplish their task.: `dialogOwner` - the owner of any Dialogs opened from the SelectorPanel (or null if there is no requested owner).


**Method Detail**

### setCenterPanelComponentsEnabled

```
public void setCenterPanelComponentsEnabled(boolean enabled)
```

:   Setting enabled to false will cause the center panel components
    to be grayed out (so they cannot be used). Setting enabled back
    to true will allow the center panel controls to be used again.

    :   **Parameters:**: `enabled` - true if components should be enabled: false otherwise.

---


### applySpinnerTilingNumber

```
public void applySpinnerTilingNumber()
```

:   Applies the tiling number in the spinner to the currently selected
    geometric object.

---


### applyDefaultTilingNumber

```
public void applyDefaultTilingNumber()
```

:   Applies the default number tiling number (slices and/or stacks)
    for the currently selected geometric object.

---


### updateSpheresLabel

```
public void updateSpheresLabel(int tiling)
```

:   Updates the tiling number text label below the "Spheres" combo
    box menu, updates the spinner value to match, and also sets the
    "Spheres" radio button as being selected. This method will be
    called by the listener for the "Spheres" combo box menu.

    :   **Parameters:**: `tiling` - the tiling number.

---


### updateCylindersLabel

```
public void updateCylindersLabel(int tiling)
```

:   Updates the tiling number text label below the 'Cylinders' combo
    box menu, updates the spinner value to match, and also sets the
    'Cylinders' radio button as being selected. This method will be
    called by the listener for the 'Cylinders' combo box menu.

    :   **Parameters:**: `tiling` - the tiling number.

---


### updateCylinderCapsLabel

```
public void updateCylinderCapsLabel(int tiling)
```

:   Updates the tiling number text label below the 'Cylinder Caps'
    combo box menu, updates the spinner value to match, and also sets
    the 'Cylinder Caps' radio button as being selected. This method
    will be called by the listener for the 'Cylinder Caps' combo box
    menu.

    :   **Parameters:**: `tiling` - the tiling number.

---


### setSpinnerToSphereValue

```
public void setSpinnerToSphereValue()
```

:   Sets the value in the tiling spinner to the value displayed under
    the radio button for spheres.

---


### setSpinnerToCylinderValue

```
public void setSpinnerToCylinderValue()
```

:   Sets the value in the tiling spinner to the value displayed under
    the radio button for cylinders.

---


### setSpinnerToCylinderCapValue

```
public void setSpinnerToCylinderCapValue()
```

:   Sets the value in the tiling spinner to the value displayed under
    the radio button for cylinder caps.

---


### toString

```
public String toString()
```

:   Returns a name suitable for use in a menu.

    :   **Overrides:**: `toString` in class `Component`
    :   **Returns:**: The menu name as a String.

---


### addAutoTilingButtonPanelToNorth

```
public void addAutoTilingButtonPanelToNorth()
```

:   This helper method for the constructor adds a check box button
    for turning automatic tiling (level of detail) on/off.

---


### addAllOtherComponentsToCenterPanel

```
public void addAllOtherComponentsToCenterPanel()
```

:   This helper method for the constructor adds the radio buttons and
    the tiling spinner to the center panel.


---


|  |  |  |
| --- | --- | --- |
| **Overview** \| **Package** \| **Class** \| **Use** \| **Tree** \| **Deprecated** \| **Index** \| **Help** | |  |
| **PREV CLASS**   **NEXT CLASS** | **FRAMES**    **NO FRAMES**     **All Classes** |  |
| SUMMARY: NESTED \| FIELD \| CONSTR \| METHOD | DETAIL: FIELD \| CONSTR \| METHOD |  |


---

# *Copyright © 2007-2008*
